# A Common Variant of PROK1 (V67I) Acts as a Genetic Modifier in Early Human Pregnancy through Down-Regulation of Gene Expression

**DOI:** 10.3390/ijms17020162

**Published:** 2016-01-27

**Authors:** Mei-Tsz Su, Jyun-Yuan Huang, Hui-Ling Tsai, Yi-Chi Chen, Pao-Lin Kuo

**Affiliations:** 1 Department of Obstetrics and Gynecology, National Cheng Kung University Hospital, College of Medicine, National Cheng Kung University, Tainan 704, Taiwan; sumeitsz@mail.ncku.edu.tw (M.-T.S.); yyuangtw@mail.ncku.edu.tw (J.-Y.H.); cuteyling@gmail.com (H.-L.T.); 2 Department of Economics, National Cheng Kung University, Tainan 704, Taiwan; yichi@mail.ncku.edu.tw

**Keywords:** modifier gene, Prokineticin 1 (PROK1; EG-VEGF), recurrent miscarriage, calcium influx, cell invasion

## Abstract

PROK1-V67I has been shown to play a role as a modifier gene in the PROK1-PROKR system of human early pregnancy. To explore the related modifier mechanism of PROK1-V67I, we carried out a comparison study at the gene expression level and the cell function alternation of V67I, and its wild-type (WT), in transiently-transfected cells. We, respectively, performed quantitative RT-PCR and ELISA assays to evaluate the protein and/or transcript level of V67I and WT in HTR-8/SV neo, JAR, Ishikawa, and HEK293 cells. Transiently V67I- or WT-transfected HTR-8/SV neo and HEK293 cells were used to investigate cell function alternations. The transcript and protein expressions were down-regulated in all cell lines, ranging from 20% to 70%, compared with WT. There were no significant differences in the ligand activities of V67I and WT with regard to cell proliferation, cell invasion, calcium influx, and tubal formation. Both PROK1 alleles promoted cell invasion and intracellular calcium mobilization, whereas they had no significant effects on cell proliferation and tubal formation. In conclusion, the biological effects of PROK1-V67I on cell functions are similar to those of WT, and the common variant of V67I may act as a modifier in the PROK1-PROKR system through down-regulation of PROK1 expression. This study may provide a general mechanism that the common variant of V67I, modifying the disease severity of PROK1-related pathophysiologies.

## 1. Introduction

Prokineticin 1 (PROK1), also known as endocrine gland-derived vascular endothelial growth factor (EG-VEGF), is a small, secreted peptide that belongs to the prokineticin family [1]. PROK1 is a tissue-specific proangiogenetic mitogen and chemotactic factor, and it acts through activation of two cognate G-protein-coupled receptors (GPCRs), prokineticin receptor 1 (PROKR1) and prokineticin receptor 2 (PROKR2). The expression of PROK1 is predominantly in the steroidogenic glands, such as ovary, testis, adrenal cortex, and placenta [2,3,4], and has been shown to have a wide range of functions including angiogenesis, modulation of inflammatory responses, and regulation of hematopoiesis) [5,6].

In recent years, PROK1 has been shown to play a role in female reproduction and human pregnancy. The temporal and spatial expression of PROK1, and its receptors in ovary and early gestational tissue, highlights their functions in follicular maturation, luteal angiogensis, embryo implantation, and uterine receptivity [1,7,8]. The dynamic expression and regulation profile of the PROK1-PROKR system through human pregnancy also suggests its regulatory role in the process of placental development and initiation of parturition [9,10,11]. Previous publication had shown that PROK1 inhibits and controls trophoblast invasion in human pregnancy [12], whereas a recent prospective study suggested PROK1 may facilitate an embryo endowing with adequate implantation potential [13]. Moreover, aberrant expression or activity of PROK1 and its receptors are also reported to be associated with several gestational complication, and most were related to inadequate or inappropriate trophoblast invasion, such as gestational trophoblastic diseases, recurrent pregnancy losses, preeclampsia, and intrauterine fetal growth restriction [11,14,15,16].

A common polymorphism of PROK1, V67I (c.199 G>A, rs7514102), located in the first nucleotide of exon 3, is a non-synonymous substitution resulting in amino acid change from valine (V) to isoleucine (I). The frequency of G to A transition varies among different ethnicities, and ranges from 43% to 64% in the general population (43% in Caucasians, 54% in Han Chinese, 64% in Japanese, and 46% in Nigerians, with this data taken from http://www.ncbi.nlm.nih.gov/pubmed/). The evolution of V67I is highly conserved, and the change of amino acid from V to I seems neutral. However, the impact of this genetic variant in the PROK1 system and its clinical relevance have not yet been explored.

We recently reported the PROK1 variant (V67I) act as a genetic modifier in human early pregnancy [17]. Previous studies of recurrent pregnancy loss (RPL) showed that women carrying PROKR1 and PROKR2 variants (I379V and V331M) have less susceptibilities for RPL risk, and that trophoblastic cell function alternation by enhancing cell invasiveness may provide the protection from recurrent abortion [17,18]. In contrast, the protection effect of the PROKR1/2 variant may be attenuated if the woman also carries PROK1-V67I [17]. Although genetic association research showed evidence of these effects, the underlying mechanism of the common variant of PROK1 (V67I) with regard to modifying RPL risk remains unclear. In order to better understand the critical role of PROK1 in physiological and pathological pregnancy, this study aimed to explore the modification role of PROK1-V67I and compare various cell functions to those of its wild-type (WT) in several associated cell lines. The results of this study may provide a general mechanism of a common variant’s effect on PROK1-related disorders.

## 2. Results

### 2.1. V67I Is a Common PROK1 Variant in the General Population

**Figure 1:**
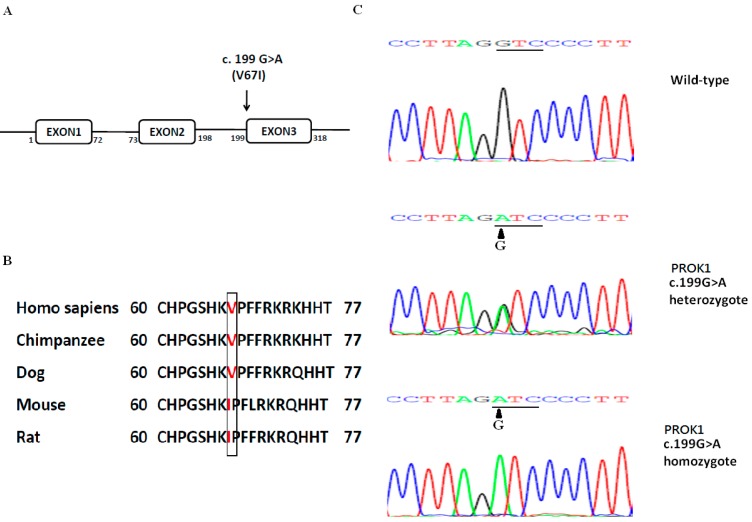
(**A**) Schematic of PROK1 variant (c.199G>A; p.Val67Ile); (**B**) PROK1 variant (V67I) is conserved in mammals; (**C**) Representative chromatographs showing the heterozygous and homozygous c.199G>A, p.Val67Ile variants and wild-type (WT) sequences.

We analyzed the coding regions of PROK1 using the Sanger sequence in 142 RPL women and 149 normal controls in a previous report [17]. The allele and genotype frequencies of PROK1-V67I variant (c.199 G>A, rs7514102) and wild-type (WT) showed no significant differences between RPL and control groups, and the pooled data are 51%, 21% and 28% for GG, GA, and AA genotypes. The highly-conserved V67I variant of PROK1 is located in the first nucleotide of exon 3, and the G to A transition changes the amino acid from Valine (V) to Isoleucine (I) (Figure 1). We further compared the population diversity of V67I using HapMap data from NCBI (http://www.ncbi.nlm.nih.gov/pubmed/). In general, the frequency of the G to A transition is around 50%, ranging from 39% to 64%, and varies among different populations (Table 1).

**Table 1:** Population diversity of PROK1 wild-type and V67I (c.199 G>A, rs7514102) variant.

Population	Group/Sample Count	Genotype Frequency			Allele Frequency	
		GG	GA	AA	G	A
HapMap-CEU	European/118	0.339	0.458	0.203	0.568	0.432
HapMap-HCB	Asian/90	0.200	0.511	0.289	0.456	0.544
HapMap-JPT	Asian/90	0.089	0.533	0.378	0.356	0.644
HapMap-YRI	Sub-Saharan African/120	0.319	0.450	0.233	0.542	0.458

Reference data of HapMap population diversity was derived from NCBI (updated 2015.6.24); GTC: Valine (V); ATC: Isoleucine (I); CEU: Utah Residents with Northern and Western European Ancestry; HCB: Han Chinese in Beijing, China; JPT: Japanese in Tokyo, Japan; YRI: Yoruba in Ibadan, Nigeria.

### 2.2. Down-Regulated Gene Expression of V67I in the Transcript and Protein Levels Compared with Wild-Type

**Figure 2:**
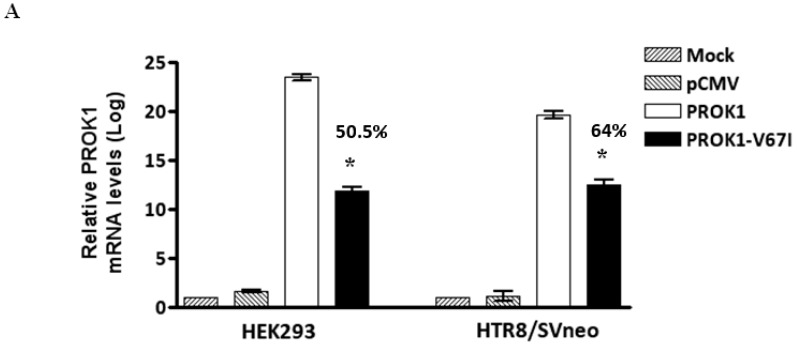

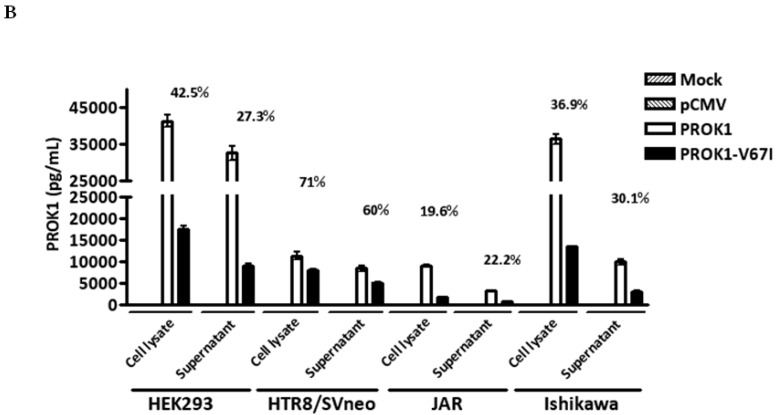
Decreased gene expression of PROK1 variant (V67I) compared with wild-type (WT) in different cell lines. Cells were transiently transfected with either WT or variant PROK1 construct for 48 h. (**A**) Quantitative RT-PCR analysis showed decreased transcript level of V67I compared with WT in cell lines (HEK293 cells: 50.5%; HTR-8/SV neo cells: 64%); (**B**) ELISA analysis showed consistently decreased protein concentrations in the cell lysate and supernatant of the culture medium among different cell lines (HEK293 cells: 42.5%–47.5%; HTR-8/SV neo cells: 60%–71%; JAR cells: 19.6%–22.2%; and Ishikawa cells: 30.1%–36.9%). The comparison of V67I and WT is shown in percentages. Data are presented as means ± SEM. * *p* < 0.001 compared with the corresponding control (WT). Mock: cells without transfecting any vectors; pCMV: cells with transfecting empty control vectors.

In order to evaluate gene expressions of PROK1-V67I and -WT, we compared different cells transfected with V67I or WT plasmids in the transcript and protein levels using qRT-PCR and ELISA assays, respectively. The transcript expression of V67I compared with WT was 50.5% in HEK-293 and 64% in HTR-8/SV neo cells (Figure 2A, *p* < 0.001). When comparing the protein levels in the cell lysate and supernatant of the culture medium, the protein expression of V67I was also down-regulated in the HEK293 (42.5%–47.5%), and HTR-8/SV neo (60%–71%) cells and showed similar results to those found for the transcript expression (Figure 2B). We further investigated the protein expression of V67I in JAR and Ishikawa cells to evaluate cell specificity, and detected lower expressions of V67I in both the cell lysate and supernatant of the culture medium in the JAR (19.6%–22.2%) and Ishikawa (30.1%–36.9%) cells (Figure 2B, *p* < 0.001). The basal protein concentration in non-transfected cells (Mock) were between 11 and 28 pg/mL in each cell line (HEK293 cells: 10.84 ± 0.85 pg/mL; HTR-8/SV neo cells: 27.93 ± 0.94 pg/mL; JAR cells: 13.77 ± 0.88 pg/mL; Ishikawa cells: 15 ± 0.38 pg/mL).

### 2.3. PROK1 Wild-Type and Variant (V67I) Have No Significantly Different Effects on Cell Proliferation and Tubal formation

**Figure 3:**
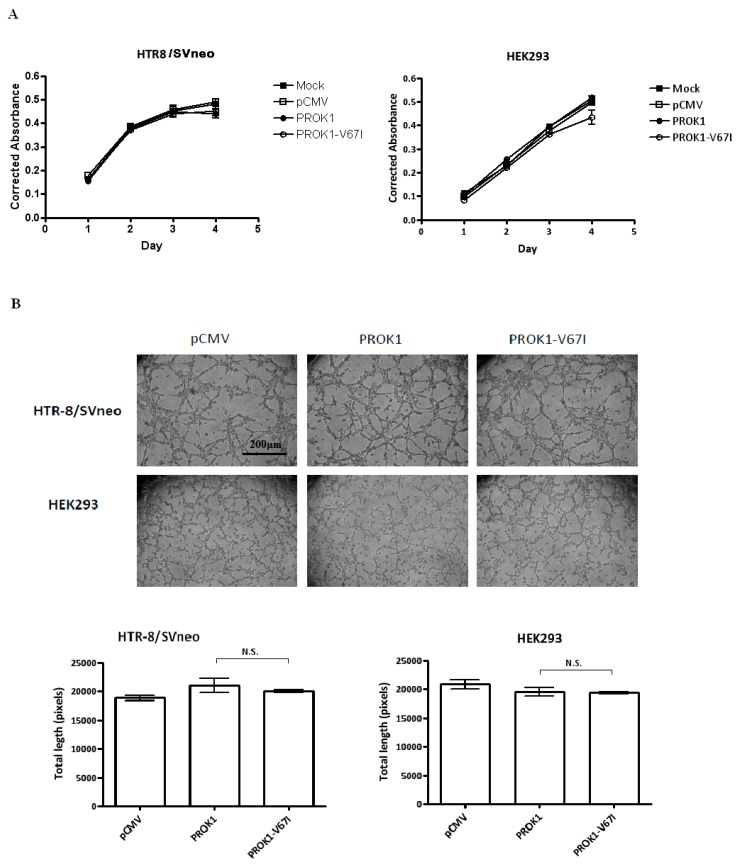
PROK1-V67I and WT did not alter cell proliferation and tubal formation. HEK 293 and HTR-8/SV neo cells were transiently transfected with control vector, WT, or variant PROK1 construct. (**A**) Cell numbers in proliferation of HEK293 and HTR-8/SV neo cells were measured, and no significant differences were detected between each group; (**B**) Photographs and quantification of tube formation in each group of HTR-8/SV neo and HEK293 cells, and no significant difference (N.S.) was detected.

Cell proliferation and angiogenesis are critical in the stages of implantation, embryogenesis, and placentation. We examined if PROK1 and its V67I variant altered the abilities of cell proliferation and tube organization, following their individual ectopic expression in cells. When comparing an empty control vector, variant, and wild-type PROK1, the cell numbers of transfected HEK293 and HTR-8/SV neo cells were not significantly different after 1 to 4 days of cell culture, based on the results of a cell viability assay (Figure 3A). To evaluate the angiogenic ability of PROK1 WT and its variant (V67I), we measured capillary tube formation of PROK1- or V67I-transfected cells by calculating branching length between two nodes at different time intervals (4–6 h). After 4 h incubation on the Matrigel, HEK-293 and HTR-8/SV neo cells rapidly reorganized and subsequently formed tube-like structures on Matrigel. Average tubal length was measured in each group, and there was no stimulatory effect on tube formation in PROK1- and V67I-transfected HEK-293 and HTR-8/SV neo cells compared with those of the empty control vector group (Figure 3B). In addition, both PROK1 and V67I groups behaved similarly, without any effects on tube formation in HEK 293 or HTR-8/SV neo cells. The results, thus, showed that PROK1 and V67I did not have any effects on cell proliferation and tubal organization in either cell line.

### 2.4. Both PROK1 Wild-Type and Its Variant (V67I) Increase Cell Invasion and Activate Intracellular Ca Influx in a Dose-Dependent Manner

**Figure 4:**
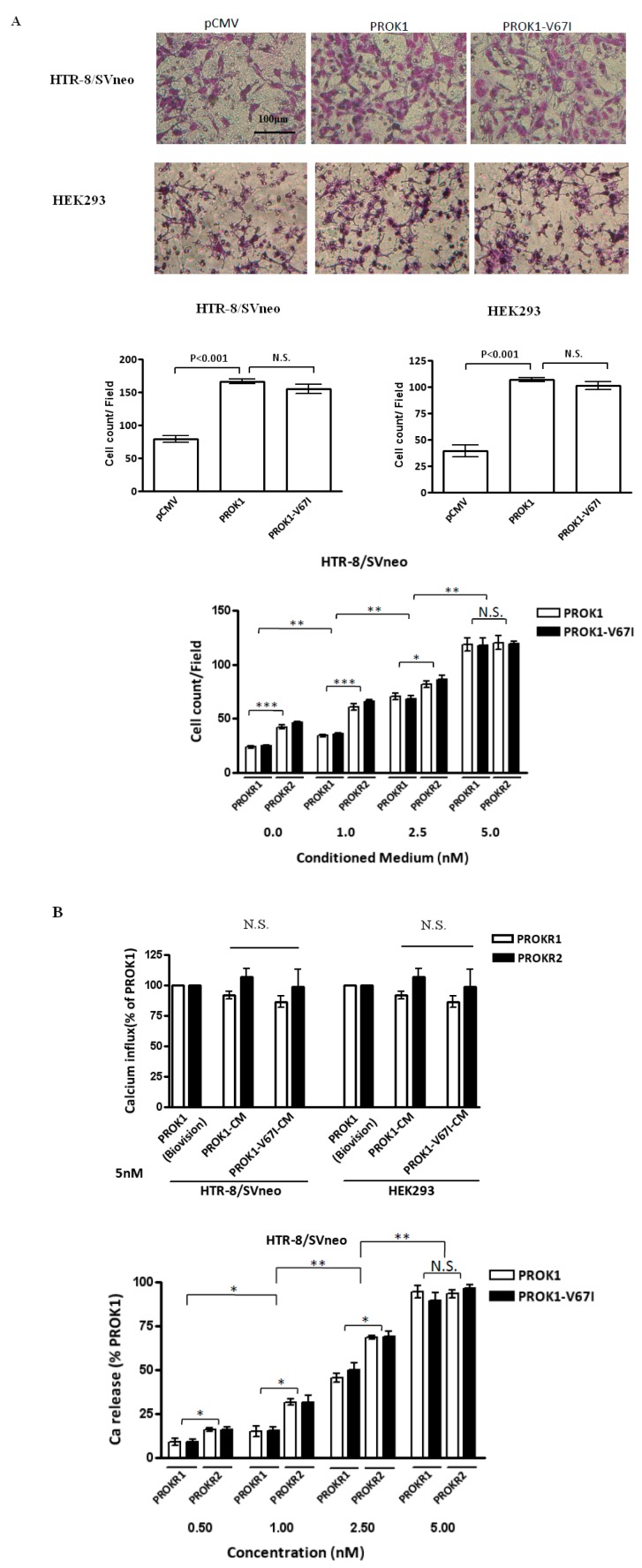
PROK1-V67I and -WT (**A**) enhanced cell invasion ability and (**B**) altered intracellular calcium influx in a dose-dependent manner. PROKR2-transfected cells had increased abilities of inducing calcium release and cell invasiveness compared with PROKR1-transfected cells. HEK 293 and HTR-8/SV neo cells were transiently transfected with control vector, WT or variant PROK1 construct. Photographs and quantification of invaded cells stained with Giemsa’s azur eosin methylene blue solution are shown in the **upper** and **middle** panels in **A**. HEK293 and HTR-8/SV neo cells were transiently transfected with either PROKR1 or PROKR2 plasmid and treated with various concentrations of PROK1 WT or V67I condition medium (0, 1.0, 2.5, 5.0 nM). Invaded cells (**A**, **lower** panel) and intracellular cell influx alternation (**B**, **upper** and **lower** panels) were measured. * *p* < 0.05; ** *p* < 0.01; *** *p* < 0.001 compared between PROKR1- and PROKR2-transfected cells, and groups of different concentration. PROK1 recombinant protein (Biovision, Milpitas, CA, USA) was used as a standard control to validate protein function of PROK1 (WT) and PROK1-V67I in condition medium (CM). N.S.: no significant difference.

In the stages of embryo implantation and further placentation, it is critical that appropriate and adequate trophoblast cell invasion is achieved. We performed cell invasion assay in a trans-well system, and found that PROK1- and V67I-expressing HEK293 and HTR-8/SV neo cells had enhanced abilities of cell invasion (Figure 4A, *p* < 0.001), but there was no difference in this enhancement between the PROK1 and V67I variant groups. We further evaluated the cell invasion ability in the prokineticin receptor 1 (PROKR1)- or prokineticin receptor 2 (PROKR2)-transfected cells, and both cell invasion abilities increased in a dose-dependent manner after treating with concentrated PROK1 and V67I (0, 1.0, 2.5, and 5.0 nM) from conditioned medium (Figure 4A, lower panel). In addition, PROKR2-transfected cells had a significantly enhanced cell invasiveness compared with PROKR1-transfected cells at 0, 1.0, and 2.5 nM of PROK1 or V67I treatment, but did not show significance at 5.0 nM (Figure 4A, lower panel).

Calcium signaling is a critical function index that is used to evaluate G protein-coupled receptors (GPCRs) and their ligand activities. We evaluated PROK1-induced intracellular calcium mobilization by using a fluorescence-based assay in the prokineticin receptor 1 (PROKR1)- or prokineticin receptor 2 (PROKR2)-overexpressed cells. In this assay, HEK293 and HTR-8/SV neo cells were initially transfected with PKOKR1 or PROKR2 plasmid to enhance the amplitude of calcium signaling before investigation. The intracellular calcium influx was stimulated, but no significant differences were seen in the PROKR1- or PROKR2-transfected cells after treating with 5 nM PROK1 or V67I from conditioned medium (Figure 4B, upper panel). Although the induced calcium influx showed a dose-dependent response after treating with PROK1 or V67I conditioned medium (0.5, 1.0, 2.5, 5.0 nM), there was no difference in the stimulation effect between PROK1 and V67I (Figure 4B, lower panel). By comparing PROKR1- and PROKR2-transfected cells on calcium signaling, PROKR2-transfected cells had a stronger signaling under concentration 0.5, 1.0, and 2.5 nM of PROK1 or V67I treatment, but did not show significance at 5.0 nM (Figure 4B, upper and lower panel).

## 3. Discussion

In a previous genetic association study of recurrent pregnancy loss (RPL), a PROK1 variant (V67I) was shown to be a genetic modifier gene in early human pregnancy [17]. We evaluated the functional effect of this common variant and compared it to PROK1-WT in order to investigate the possible mechanism of genetic modification in this study. We demonstrated that the conserved non-synonymous variant of PROK1 (V67I) has similar cell functions with regard to enhancing trophoblast cell invasion and stimulating intracellular calcium signaling compared with its wild-type. However, the gene expression of V67I is down-regulated in both transcript and protein levels across all associated cell lineages.

The biological interaction of PROK1 and PROKR1/2 is a ligand-receptor relationship. The impact of an amino acid change in PROK1 on the protein activity and the binding affinity between its receptors remain unclear, and no PROK1 variants have been reported before. We identified PROK1-V67I to be the only non-synonymous variant in the coding regions of PROK1 using Sanger sequence in 291 subjects (142 RPL patients and 149 normal controls) [17]. The non-synonymous variant of PROK1-V67I is so common that it is possessed by nearly 50% of the general population, appears to have no visible effect on the fitness of individuals and is, therefore, usually considered as a neutral variant. However, several studies have demonstrated that a common non-synonymous variant may have an association with or functional significance for the pathophysiology of a disease [19,20,21]. From an evolutionary perspective, the common non-synonymous variants usually have modest structural effects, and some of them are simply functionally neutral, while others are deleterious [22]. Moreover, some variant sites that are predicted to be deleterious might have advantages under unique conditions of developmental or environmental changes [22]. During the early stages of placental development, the coordination of cell proliferation, differentiation, and invasion of trophoblasts is subtle and fine-tuned in the feto-maternal interface. The lower protein production of V67I seems to have a negative effect on the function of PROK1, which implies less cell invasion ability and attenuated downstream calcium signaling. However, whether the down-regulated PROK1 expression is beneficial or deleterious is an issue that requires more research.

The PROK1 level during pregnancy is dynamic, both in placental tissue and serum collected from pregnant women. Placental PROK1 expression gradually rises after embryo implantation and peaks at 8–11 weeks of gestation, subsequently decreasing until the end of the first trimester, and then this level is maintained throughout the remaining gestation [4,7,23]. Plasma PROK1 concentration also gradually increases during the first trimester (~200 pg/mL), and then decreased to ~70–80 pg/mL in the second and third trimesters [23]. Adequate trophoblast invasion and proper placental development ensures the embryo or fetus has sufficient nutrient and oxygen, and PROK1 is regarded as playing a critical role in regulating trophoblast invasion and placental development [13,23]. Excessive trophoblast invasion could result in placenta accreta and invasive moles, whereas poor trophoblast invasion of maternal vessels could contribute to preeclampsia and intrauterine fetal growth restriction [10,13,23,24]. As a result, the differential down-regulated PROK1 expression of V67I in different cell lineages presented in this study could be an underlying cause for a pathological condition, or a normal regulatory mechanism of the PROK1 system in human pregnancy. We, therefore, speculated that women carrying different PROK1 genotypes may have varied susceptibility to different pregnancy complications. Moreover, PROK1 was shown to be up-regulated in several types of cancers, such as colorectal cancer, pancreatic cancer, prostate cancer, and ovarian cancer [25,26,27,28,29,30], and was regarded as a poor prognostic marker and survival factor. These PROK1-related malignancies were closely associated with advanced clinical stage, histological grade, and distant metastasis, which may probably through regulating peritumoral angiogenesis or/and strengthened cancer cell invasiveness [25,26,27,28,29,30]. The PROK1-overexpressing cells in the present study could be a mimicking condition of malignancy, and the increased cell invasion ability suggested the potential role of PROK1 in cancer biology. Nevertheless, more studies are required to elucidate the role of PROK1 WT and V67I in both human pregnancy and cancers, and the complex interaction between PROK1 and its receptors in various pathophysiologies of human clinical situations.

Disease severity could be influenced by different genetic backgrounds, and genetic modifiers are known to alter the outcomes in various human diseases or animal models of disease, such as muscular dystrophy (MD), epidermolysis bullosa (EB), and some motor neuron diseases [31,32,33,34,35]. Since genetic modifiers act in a non-Mendelian manner to alter the phenotype in question, the comparatively small effect of V67I in the general population is highly consistent with its role as a modifier in early human pregnancy. From our previous data, PROKR1 and PROKR2 variants (I379V and V331M) may protect women from RPL through enhanced trophoblast invasiveness, thus facilitating embryo implantation [18]. In women carrying wild-types of PROKR1 and PROKR2, the genotypes of PROK1 WT or V67I do not have significant effects on RPL risk. In contrast, women carrying V67I will lose the protective effect with regard to RPL risk if they also carry PROKR1 or PROKR2 variants, indicating that PROK1-V67I modifies RPL risk in specific populations [17]. This could be explained by the results of the cell functional assays carried out in the present study. These showed that the effects of V67I on cell function are not different from those of its wild-type, although its gene expression efficiency is only 20%–70% that of the latter (depending on cell types). The ability of trophoblast invasion is promoted by PROK1 and V67I, and both facilitate invasiveness in a dose-response manner. Therefore, PROK1-V67I may reduce PROKRs’ protective effect of promoting cell invasiveness by decreasing PROK1 protein production.

## 4. Experimental Section

### 4.1. Subjects

The present study was approved by the Institutional Review Board of National Cheng Kung University Hospital (#HR-96-39) (Tainan, Taiwan), and informed consents were obtained from all patients and controls. The clinical and molecular details of the RPL subjects examined in the current work were reported in a previous study [17,18]. In this study we compared the *V67I* allele and genotype frequencies of our population, Han Chinese individuals in Taiwan, with those of other HapMap Populations (Table 1).

### 4.2. Cell Cultures and Treatments

The human HTR-8/SVneo trophoblast cell line was a gift from Dr. Charles Graham (Queen’s University, Kingston, ON, Canada). HTR-8/SVneo cells were grown in RPMI 1640 medium (Invitrogen, Grand Island, NY, USA) supplemented with 10% fetal bovine serum (FBS) and 100 IU/mL penicillin-streptomycin. The human embryonic kidney cell line (HEK293) were grown in Dulbecco Modified Eagle’s Medium (DMEM) (Invitrogen) supplemented with 10% FBS (Invitrogen) and 1% penicillin-streptomycin solution. JAR cells were derived from human placental choriocarcinoma, which were purchased from Bioresource Collection and Research Center (Taiwan, Taiwan), and grown in RPMI 1640 medium with 10 mM HEPES, 1 mM sodium pyruvate, 10% FBS and 1% penicillin-streptomycin solution. Ishikawa cells were grown in Minimum Essential Media (MEM) (Invitrogen) supplemented with 1% non-essential amino acids (NEAA) + 5% FBS and 1% penicillin-streptomycin solution. These cells were cultured in a 5% CO_2_ humidified incubator at 37 °C. After confluent growth, the attached cells were trypsinized and either cryopreserved or subcultured for further use.

### 4.3. Generation of Variant PROK1 Expressing Plasmids and Transfection Experiments

The variant sequence of PROK1 (V67I) was introduced into the WT Myc-DDK-tagged cDNA in a pCMV6-Entry vector (Origene Technologies, Inc., Rockville, MD, USA), which encodes the entire coding regions of human cDNA (GenBank NM_032414.2) of PROK1, using Stratagene’s Quik Change II Site-directed Mutagenesis Kit (La Jolla, CA, USA). A vector control, pCMV6 empty vector, was constructed from pCMV6-PROK1 plasmid using sgfI and MluI digestion and the T4 ligation method. All constructs were verified by nucleotide sequencing. pCMV6 and PROK1 constructs were propagated in JM109 Escherichia coli. Transfection efficiency was tested on HEK293 (5 × 10^5^ cells) and HTR8/SVneo (3 × 10^5^ cells) using 1 μg of pCMV-EGFP (Clontech Laboratories, Inc., Palo, Alto, CA, USA) expressing plasmid and 3 µL of TurboFect in six-well plates for 24 and 48 h. The transfection efficiency is 90% ± 10% in the two cell lines. The established constructs were transfected into HEK293 and HTR-8/SVneo cell lines by Turbofect for 24 h (Fermentas/*Thermo Scientific*, Waltham, MA, USA), and their expressions were confirmed by Western blotting. The PROKR1 and PROKR2 plasmids were constructed as reported in a previous study [18].

### 4.4. Measurements of PROK1 Gene Expression

#### 4.4.1. Quantitative Real-Time PCR (qRT-PCR) Analysis

We extracted total RNA from scraped cells using the Trizol reagents (Invitrogen, Carlsbad, CA, USA) according to the manufacturer’s instructions. The extracted RNA was spectrophotometrically quantified, and its quality was assessed by measuring the absorbance ratios at 260/280 and 260/230 nm using a GeneQuantTM Pro Spectrophotometer (GE Healthcare Biosciences, Piscataway, NJ, USA). Two micrograms of total RNA was reverse-transcribed using a SuperScript^®^ Reverse Transcription kit (Invitrogen, Carlsbad, CA, USA) RNaseOut, dNTPs and random primers according to the manufacturer’s protocols. The qPCR reactions were carried out in an Applied Biosystems StepOne Plus system (Applied Biosystems, Foster City, CA, USA). The primers used were the PROK1 sense primer, 5’-CATGCTCCTCCTAGTAACTG-3’, the PROK1 antisense primer, 5’-TTTCCTGAAGAAGGGGAC-3’, and the internal reference, GAPDH sense primer, 5’-ACAGTTGCCATGTAGACC-3’, and the GAPDH antisense primer, 5’-TTTTTGGTTGAGCACAGG-3’, which amplifies fragments of 190 bp for PROK1 cDNA and 225 bp for GAPDH cDNA (Sigma-Aldrich, Bornem, Belgium). A 10-μL reaction mixture containing cDNA, specific primers and Fast SYBR Green^®^ Master Mix (Applied Biosystems, Carlsbad, CA, USA) was used in the PCR. Reactions were performed at 95 °C for 20 s, followed by 40 cycles of 95 °C for 3 s and 60 °C for 30 s. To calculate the relative expression for each gene, the 2^−ΔΔ*C*t^ method was used to relate the *C*_t_ values of PROK1 expression in each sample to the *C*_t_ values of GAPDH.

#### 4.4.2. Immunoassay (ELISA)

HEK293, HTR-8/SVneo, JAR, and Ishikawa cells (3 × 10^5^) were transfected with pCMV vector, PROK1 wild type or V67I plasmid for 48 h. Cell culture supernatant and cell lysate (cells were lysed with RIPA buffer) were harvested for an assay using an ELISA kit to assess the PROK1 concentration following the manufacturer’s instructions (R and D Systems, Minneapolis, MN, USA). Briefly, supernatant or cell lysate were applied to the microplate coated with the capture antibody for 2 h at room temperature. Detection antibody conjugated to streptavidin-horseradish peroxidase was then applied, followed by the color development solution (tetramethylbezidine substrate) for 20 min. Color development was terminated by addition of sulfuric acid, and the optical density was determined at 450 nm by a microplate reader (SpectraMa × 340PC384, Molecular Devices, Sunnyvale, CA, USA). A PROK1 standard (Biovision, Milpitas, CA, USA) was used for control, and calculation of the results was performed using computer software capable of generating a four parameter logistic (4-PL) curve-fit.

### 4.5. Recombinant PROK1 and V67I by Concentrating Secreted Proteins

HEK293 cells (5 × 10^5^) were transfected in six-well plates with PROK1-WT or PROK1-V67I plasmid in DMEM with 1% FBS for 48 h. We then collected the culture medium to concentrate secreted protein using a centrifugal filter device, Amicon^®^ Ultra4-3000 MWCO (Merck Millipore, Tullagreen, Carrigtwohill, Co., Cork, Ireland), under 3500 rpm for 40 min. We calculated the concentrated protein level using the ELISA method, as described above. The concentrated protein medium (conditioned medium (CM)) was stored at −80 °C, and its protein activity (WT and V67I) was compared with that of a PROK1 standard (Biovision, Milpitas, CA, USA) and tested for various cell functions, as described below.

### 4.6. Cell Proliferation Assay

The effect of PROK1 variant (V67I) on cell proliferation was assessed when compared with that of PROK1 wild-type and pCMV6 control. Cell lines were grown at 2 × 10^3^ cells/well in a 96-well plate in DMEM or RPMI medium containing 10% FBS for 24 h. Cell proliferation was determined after one, two, three, and four days. Cell numbers were determined with PrestoBlue™ cell viability reagent (Invitrogen, Carlsbad, CA, USA). After adding 100 μL of cell proliferation reagent in each well for 6 h at 37 °C in a CO_2_ incubator, the absorbance at 570 nm (with 600 nm as the reference wavelength for normalization), reflecting the number of viable cells, was measured with a microplate reader (SpectraMax 340PC^384^, Molecular Devices, Sunnyvale, CA, USA). All the treatments were carried out in quadruplicate, and each experiment was carried out at least three times.

### 4.7. Tube Formation Assay

The effects of PROK1 wild-type and variant (V67I) on tube organization were assessed by growing transfected cells (HEK293 and HTR-8/SVneo) on Matrigel. Approximately 80 μL ice-cold Matrigel (BD Biosciences, San Diego, CA, USA) was layered into each well of a 96-well plate. The Matrigel was allowed to completely solidify at 37 °C for 1 h. The transfected HEK293 (5 × 10^4^ cells/well) and HTR-8/SVneo (3.5 × 10^4^ cells/well) cells were added and incubated at 37 °C in an atmosphere of humidified 95% air/5% CO_2_ for 4 and 6 h, respectively. Observations were made under an inverted photomicroscope to document the developmental stages. Tubal formation was assessed by measuring tubal length in four quadrants of high power fields in each well. Each assay was done in triplicate and each experiment was repeated at least three times. Quantification was measured with Image J software (Image J 1.47 h, Wayne Rasband, National Institute of Mental Health, Bethesda, MD, USA).

### 4.8. Cell Invasion Assay

To investigate the effects of PROK1 and its variant (V67I) on cell invasion, the transfected HEK293 (1.5 × 10^5^) or HTR-8/SVneo (1 × 10^4^) cells were trypsinized and re-suspended in serum-free medium and placed in the upper chamber coated with Matrigel (1 mg/mL at 37 °C for 1 h; BD Biosciences, San Diego, CA, USA) in trans-well plates (millicell cell culture insert; 8 μm pore size; Milipore, MA, USA) with or without PROK1 or V67I conditioned medium (1.0, 2.5, 5.0 nM). DMEM containing 10% FBS was placed in the lower chamber. The cells were incubated for 20 h in a humidified atmosphere with 95% air and 5% CO_2_ at 37 °C. Invaded cells on the bottom side of the membrane were fixed with 100% cold methanol and stained with Giesmsa’s azur eosin methylene blue solution (Merck, Darmatadt, Germany) and counted from a minimum of four high-power fields per insert under light microscopy. The data were expressed as the averages of three independent experiments.

### 4.9. Intracellular Calcium Influx Assay

Twenty-four hours after transfection, cells were harvested from plates using EDTA-trypsin, and washed with HBSS-based buffer (20 mM HEPES, 1 mM MgSO_4_, 3.3 mM Na_2_CO_3_, 1.3 mM CaCl_2_, 2.5 mM probenecid, pH 7.4) supplemented with 0.1% bovine serum album. Cells were loaded with 4 μM calcium indicator Fluo-4 AM (Molecular Probes, Eugene, OR, USA) for 1 h at 37 °C. After washing twice, the cells were re-suspended to a concentration of 1 × 10^6^ cells/mL. The green fluorescence emission of Fluo-4 was analyzed using FACSCalibur flow cytometry (BD Immunocytometry System), as described previously [18]. Following the establishment of a green fluorescence Ca^2+^ baseline, the indicated level of concentrated PROK1, V67I or PROK1 standard (Biovision, Milpitas, CA, USA) (0.5, 1, 2.5, or 5 nM) was added to the cell suspension to detect fluctuations in the green fluorescence.

### 4.10. Statistical Analysis

All values of the experimental assays were expressed as means ± SEM. Differences between the groups were compared using the unpaired two-tailed *t*-test or one-way ANOVA, and a *p* value of less than 0.05 was considered statistically significant.

## 5. Conclusions

We evaluated the function and possible regulatory mechanism of a common variant of PROK1 as a genetic modifier in early human pregnancy. Although PROK1-V67I has a similar functional effect to that of WT on cell behavior, gene expression at the transcript and protein levels is impaired. The results of this work provide an explanation for how a common, innocuous PROK1 variant could act epistatically with its receptor genes to interfere with the outcome of early human pregnancy. However, more research is needed to assess whether the clinical impact of the decreased protein production of PROK1-V67I is positive or negative. Considering the important role of PROK1 in pregnancy and the high prevalence of V67I in the general population, the findings of the present study may provide a common mechanism with regard to modulate the risk of various PROK1-related diseases.
